# Effects of boat noise on fish fast-start escape response depend on engine type

**DOI:** 10.1038/s41598-019-43099-5

**Published:** 2019-04-25

**Authors:** Mark I. McCormick, Eric P. Fakan, Sophie L. Nedelec, Bridie J. M. Allan

**Affiliations:** 1 ARC Centre of Excellence for Coral Reef Studies, and Department of Marine Biology and Aquaculture, James Cook University, Townsville, Queensland 4811 Australia; 2 Biosciences, College of Life and Environmental Sciences, University of Exeter, Geoffrey Pope, Stocker Road, Exeter, EX4 4QD UK; 3 Present Address: Department of Marine Science, University of Otago, Dunedin, 9054 New Zealand

**Keywords:** Population dynamics, Animal behaviour

## Abstract

Vessel noise represents a relatively recent but rapidly increasing form of pollution, which affects the many organisms that use sound to inform their behavioural decisions. Recent research shows that anthropogenic noise can lead to reduced responsiveness to risk and higher mortality. The current laboratory experiment determined whether the playback of noise from motorboats powered by two- or four-stroke outboard engines affected the kinematics of the fast-start response in a juvenile coral reef fish, and the time scale over which the effects may occur. Results show that the two engine types produce slightly different sound spectra, which influence fish differently. Playback of 2-stroke engines had the greatest effect on activity, but only for a brief period (45 s). While noise from 4-stroke outboard engines affected fast-start kinematics, they had half the impact of noise from 2-stroke engines. Two-stroke engine noise affected routine swimming more than 4-stroke engines, while 4-stroke noise had a greater effect on the speed at which fish responded to a startle. Evidence suggests that the source of the noise pollution will have a major influence on the way marine organisms will respond, and this gives managers an important tool whereby they may reduce the effects of noise pollution on protected communities.

## Introduction

Vessel noise is recognised as an important source of pollution that is ubiquitous in aquatic environments within populated areas around the world^1,2^. Currently, there are over 9.3 million registered small (<8 m) motorboats in the USA^3^, 6 million in Europe^4^ and over 90,000 recreational motorboats on the Great Barrier Reef, Australia^5^. The impact of small motorboats has not received as much attention as the 94,000 merchant ships greater than 100 gross tonnes that traverse the oceans of the world^6^. However, small motorboats can potentially have a significant impact on communities of marine organisms, as motorboats use much of the shallow waterways and coastline that form the most productive habitats and sustain important fisheries.

Anthropogenically-produced marine noise has been found to have significant effects on the distribution, behaviour, reproduction and survival of marine invertebrates and fishes^7,8^. These organisms often use sound as an important source of information and have well-developed ways of producing and hearing sound^9,10^. Many fishes, such as various species of perciform fishes (which comprise about 41% of all bony fishes^11^), use sound for communication during territorial defence, aggression, mating, and as a method of indicating alarm^12^. Noise from vessels can mask, disrupt or distract organisms that use sound as a source of information^1^. While there has been concern at the level of some governments to limit anthropogenically-produced noise within inshore areas through legislation^13^, the empirical foundation on which these policies are based is generally lacking^14–16^.

One of the most disruptive effects that anthropogenic noise can have on fishes is through its effect on their ability to assess risk and make choices concerning anti-predator behaviour (e.g.^17,18^). This is particularly the case at the end of the larval phase when reef fishes, like other demersal marine organisms, are particularly vulnerable to predation. This life stage represents a population bottleneck, with high and variable mortality rates, averaging 50% within 2d of settlement^19^. Anything that alters the mortality trajectories at this early life stage may have a marked influence on the numbers entering subsequent less vulnerable life stages. Recently, Ferrari *et al*.^20^ found that 2-stroke motorboat noise affected the ability of settlement-stage fish to learn the identity of novel predators, leading to a 70% decrease in survival in the wild over 72 h. Simpson *et al*.^17^ also found that noise from motorboats reduced the likelihood and initiation distance of appropriate escape responses in juvenile fish. Short-term effects of anthropogenic noise on cognition and behaviour could therefore have a critical influence on survival via impacts on risk assessment, decision-making and anti-predator behaviour. However, detailed investigation of the kinematics of anti-predator responses in response to noise are still lacking.

When startled by a predator, fishes undertake a fast-start that involves a contraction of the muscles on one side of the body (into a C-bend) controlled by large Mauthner neurons, followed by the rapid contraction of the muscles on the opposite side leading to a rapid burst (~0.1 s) away from the original position^21^. The importance of performance in relation to a startle stimulus was recently emphasised by a study that assessed the ability of 18 morphological, behavioural and performance traits to predict the survival of juvenile damselfish in the field. McCormick *et al*.^22^ found that latency to respond, measured in the same way as the present study, was one of five key predictors of survival, the others being aspects of behaviour and space use in the field.

Knowing whether particular types of motors, which produce noise with specific characteristics (e.g., frequency and power spectra), have different effects on fish performance is useful as it gives marine managers options for the regulation of marine noise. Two-stroke outboard engines power much of the inshore fleet of small motorboats used by recreational boaters around Australia and the Pacific nations due to their low cost and high power-to-weight ratio. Some countries have effectively outlawed their use due to the high smoke emissions (e.g., USA) in preference for 4-stroke engines^23^. To date however, few studies have examined the relative merits of 2-stroke and 4-stroke outboard engines as sources of marine noise and how their noises may differentially affect marine organisms.

The goal of the current study was to determine whether noise from motorboats powered by two- or four-stroke outboard engines affected the routine swimming and kinematics of the fast-start response in a juvenile coral reef fish, and the time scale over which the effect may occur. Our prediction, based on previous research (e.g.^17,18^) was that noise from 2-stroke outboard engine would be more detrimental to the fish than 4-stroke engines due to differences in the sounds produced.

## Materials and Methods

### Study species

Our study focused on individuals that were young-of-the-year, and specifically at the end of the larval phase as they settled to join the reef-associated juvenile population^24^. This is when fish suffer high levels of selective mortality from reef-based predators^19,25^, which can constrain the phenotypic and genetic variability entering juvenile and adult life stages^26,27^. Any stressor, such as noise, that affects the likelihood of survival at this life-history bottleneck will have repercussions for later life stages. The whitetail damselfish, *Pomacentrus chrysurus* (Pomacentridae), was chosen as the target species for the study because it has life-history characteristics that are typical of many reef fishes, and young-of-the-year were caught in sufficiently large numbers for a well replicated experiment. It is a rubble-associated planktivore commonly found across the Indo-Pacific^28^. Newly metamorphosed whitetail damselfish were collected with light traps moored at least 50 m off the fringing reef of Lizard Island (14°40′12.13″S, 145°27′42.20″E), northern Great Barrier Reef, Australia in February 2017. On the morning of capture, whitetail damselfish were transferred to 30 L tanks and fed *Artemia* in excess of requirements twice daily. Tanks did not have aeration stones and fresh seawater entered the tank through a pipe below the surface to reduce noise (Fig. S1). Juveniles were held for 1 to 2 d prior to use in experiments to allow recovery from the stress of capture, at which time they were 12.1–15.6 mm total length (mean ± SD; 13.92 ± 0.73 mm). They were not fed for 12 h prior to commencement of the experimental trials to standardise for satiation. Temperature was monitored throughout the study period (range 27.8 to 29.7 °C) and treatments were systematically undertaken throughout each day.

### Ethics statement

All methods and research within this study were carried out in accordance with the animal ethics guidelines and regulations of the James Cook University, and all protocols were approved by the James Cook University Animal Ethics Committee (Approval Numbers: A2080 and A2408).

### Soundscapes

Playback tracks of ambient reef noise and 2- and 4-stroke engines were composed from recordings made in October 2016 using a calibrated omnidirectional hydrophone (HTI-96-MIN; frequency response 2–30,000 Hz, voltage sensitivity −165 dB re 1 V/µPa; High Tech, Inc., Gulfport, Mississippi) and a digital recorder (Sony PCM-M10, 24-Bit, 48 kHz sampling rate; Sony Corporation, Tokyo, Japan) which was also fully calibrated using pure sine wave signals, measured in line with an oscilloscope, and produced by a function generator (TTi RS Components 216-069, TG230, 2 MHz Sweep/Function Generator). The hydrophone was held 2 m from the surface by a researcher working from a small kayak to eliminate the noise of waves slapping on the hull of a research boat. Ambient reef sound was recorded next to a reef in 5 m water depth. The boat noise treatments were recorded at the same location with five 2-stroke and four 4-stroke engines on 5 m long dinghies each with a 30 hp motor driving past the hydrophone (20 to 100 m away). Outboard engines were 30 hp Suzuki DT30 (2-stroke) and DF30A (4-stroke).

Sound recordings were made for all parts of the experimental process from transport from the light traps, and maintenance within holding tanks (Fig. S1), through to exposure noise from the experimental motorboats. For all recordings, pressure was recorded using the hydrophone described above and particle motion, the component of sound heard by all fish^29^, was also measured using an accelerometer (M20-040, frequency response 0–3000 Hz, with a sensitivity curve over its response range, calibrated by manufacturers, Geospectrum Techologies, Dartmouth, Canada) and a digital recorder (Zoom). Sound recordings of both the field and tank recordings were analyized with paPAM acoustics analysis package (v9.3; see^24^; Fast-Fourier Transformation length = 1024, Hamming window with 50% overlap between 0–2 kHz).

Differences between acoustic pressure emitted by the two engine types were explored using five recommended acoustic metrics: Root-Mean-Square sound pressure level (dB re 1 uPa) (RMS); Peak level (dB re 1 uPa); Consistency (percent time sound level exceeded 150 dB re 1 uPa); 90% energy envelope (time for 90% of the sound energy to be emitted); and Acoustic Complexity Index (ACI; quantifies the variability in the intensity of sounds)^30–32^. Ten separate playback recordings for 2- and 4-stroke engines were collected (5 different engines for 2-stroke and 4 different 4-stroke) and analysed with MATLAB 2013a using paPAM analysis package and r packages “Soundecology” (v1.3.3) and re-verified with “seewave” (v2.1.0). To examine the noise within a biological context, a bandwidth between 0–2 kHz was applied to the analysis because pomacentrids are known to detect sounds within this range^33^.

### Experimental treatments

Two days after capture in light traps, fish were placed into individual 1 L aquaria and left in a water bath with subdued light for 30 to 60 min prior to their use in the test arena. Individual fish were then transferred to the fast-start arena (Fig. S2) and allowed to habituate for 5 min under a sound recording of daytime ambient reef noise. Fish were then either given ambient reef sound for a further 5 min or exposed to noise from one of two types of motorboats (powered by 2-stroke or a 4-stroke 30 hp engine) for 5 min. Four tracks of each noise treatment were created by splicing 1 min sections of 5 ambient or 5 boat passes together. The noise treatments were maintained for a further 2 minutes, or if ambient they may have then received one of the boat noise treatments (representing the acute noise-treatments; see Table 1). This resulted in 5 acoustic treatment combinations: (a) ambient reef sound; (b) acute 2-stroke engine noise; (c) acute 4-stroke engine noise; (d) chronic 2-stroke engine noise; (e) chronic 4-stroke engine noise (Table 1

**Table 1:**
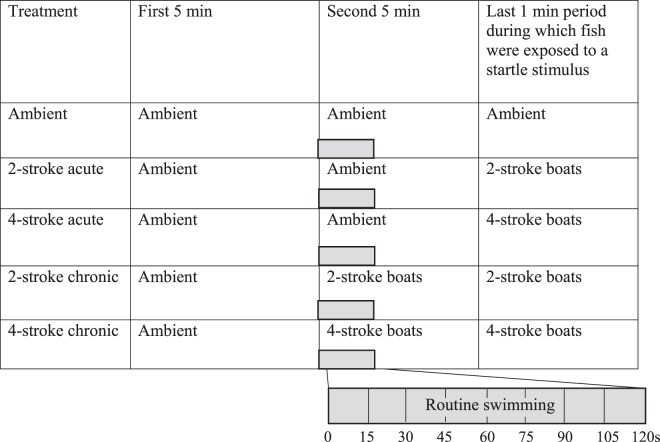
Noise treatments used to determine the effects of motorboat noise on the fast-start kinematics of the white-tail damselfish, *Pomacentrus chrysurus*. Fish were exposed to the playback of ambient reef sound, or the noise from the passage of small boats powered by 30 hp 2-stroke or 4-stroke outboard motors. Fish were startled by a drop stimulus as soon as they got into an appropriate position away from the walls of the arena after 10 min.

). The terms acute and chronic are simply used in a relative sense here, with the design used to determine the extent to which the findings are caused by a rapid change in acoustic environment rather than differences in the acoustics among the three sound types (ambient, 2-stroke, 4-stroke) themselves.

### Fast-start arena and protocol

The fast start arena consisted of a thin (1.5 mm) transparent plastic circular arena (diameter 20 cm) within a 60 L white-sided container (35 × 40 × 25 cm, Fig. S2) that had a Perspex bottom. The tank was filled with seawater (~40 L) so that the speaker, which was positioned near but not touching one wall was completely covered. Holes in the inner arena allowed flow of aerated water during habituation. The water depth within the fast-start arena was 6 cm, which restricted movement in the vertical plane, and this was achieved by placing the arena on a Perspex table (Fig. S2). The whole holding tank was illuminated by an LED light strip wrapped around the outside of the tank with light penetrating with even illumination through the white plastic sides. The tank was covered with a white plastic board (Corflute) to simplify the video background. Fast start responses were elicited by the release of a tapered metal weight from above the water surface. This was accomplished by turning off an electromagnet which held the weight. The drop distance of the metal weight was controlled by a piece of nylon line that was just long enough to allow the tapered tip to touch the water surface. In order to provide a sudden stimulation and allow calculation of the escape latency, the stimulus was released through a white PVC tube (diameter 48.5 mm) suspended above the experimental arena and passing through the lid, with the bottom edge at a distance of 10 mm above the water level. The startle stimulus was released when fish were 2 to 2.5 body lengths off the wall (~35 mm), allowing an individual to move an equal distance in any direction and standardising for fish position relative to the stimulus. Escape responses were recorded at 480 fps (Casio EX-ZR1000) as a silhouette from below obtained through pointing the camera at a mirror angled at 45°. Prey escape variables were only measured when prey performed a C-start. Only 13 fish failed to undertake a C-start across all treatments. Treatment trials were systematically conducted between 8:00 and 16:00 h.

### Routine swimming

At the start of the second 5 min period within the fast-start arena, fish were videoed at 30 fps for 2 min to obtain an estimate of their space use and behaviour while under ambient or boat noise (2- and 4-stroke engines). The following variables were recorded:

1. Thigmotaxis, recorded as time out of 120 s spent within 2.5 average body lengths (i.e., 35 mm) off the wall of the arena.
2. Total distance moved was calculated by tracking an individual’s position every 1 s for a period of 120 s. To examine if this variable changed with time, the data was separated into 15 s intervals.

### Kinematic variables

Kinematic variables associated with the fast-start response were analysed using the image-analysis software Image-J, with a manual tracking plug-in (imagej.nih.gov/ij/). The centre of mass (CoM) of each fish was tracked for the duration of the response. The following kinematic variables were measured:

1. Response latency (s) was measured as the time interval between the stimulus onset and the first detectable movement leading to the escape of the animal.
2. Response distance (m) is a measure of the total distance covered by the fish during the first two flips of the tail (the first two axial bends, i.e. stages 1 and 2 defined based on Domenici and Blake^21^, which is the period considered crucial for avoiding ambush predator attacks^34^.
3. Response speed (m/s) was measured as the distance covered within a fixed time (25 ms). This fixed duration was based on the average duration (22.8 ms) of stage 1 and 2 (as defined above).
4. Maximum response speed (m/s) was measured as the maximum speed achieved at any time during stage 1 and stage 2.

In addition, distance of the fish’s centre of mass to the stimulus at the point of contact with the water surface was recorded to be used as a covariate when applicable. Most variables measured have been previously posited to be related to fish fitness (Table 2).

**Table 2:** Variables measured for each juvenile white-tailed damselfish. Descriptive statistics for the 261 fish used are given together with their coefficient of variation (CV).

Variable type	Variable	Mean	SD	Min, Max	CV	Transformation	Reference
Morphological	Standard length (SL, cm)	13.90	0.73	12.1 15.6	5.3	Nil	^51^
Kinematic	Response latency (s)	0.017	0.012	0.006, 0.11	72.4	x^−0.5^	^52^
	Response distance (m)	0.021	0.003	0.012, 0.027	12.3	Nil	^53^
	Response speed (m/s)	0.855	0.105	0.472, 1.090	12.3	Nil	^54^
	Maximum response speed (m/s)	1.200	0.176	0.711, 1.717	14.7	Nil	^53^
Routine swimming	Distance moved (m)	1.418	1.071	0.193, 8.579	75.6	Log_10_(x)	^54^
	Average speed (m/s)	0.012	0.009	0.002, 0.072	75.6	Log_10_(x)	^55^
	Maximum speed (m/s)	0.045	0.026	0.026, 0.149	57.4	Log_10_(x)	

References that postulate the importance of the variables to fitness are given.

### Statistical analyses

A multivariate analysis of variance (MANOVA) was undertaken to determine whether there was a difference in the routine swimming or fast start kinematics among the playback of ambient sound, 2-stroke and 4-stroke engine noise treatments. To allow the routine swimming measures and kinematics to be incorporated into the same analysis, only the fish exposed to chronic sound treatments (Table 1) were used in this comparison. Pairwise, sequential Bonferroni corrected MANOVAs were used to determine the nature of differences between treatments. Multivariate normality (quantile-quantile plots) and homogeneity of covariance matrices (Box M test) were examined and variables were transformed to conform to normality (Table 2). Response latency was affected by the distance to the drop stimulus and this was found to be a significant covariate, though was consistent among treatments (satisfying the assumption of homogeneity of slopes). A linear relationship between response latency and distance to the drop stimulus was addressed by using the residuals of the relationship (both square-root transformed to improve normality) in the MANOVA. The nature of significant differences found by MANOVA in relation to the original variables was displayed using a canonical discriminant analysis (CDA). This analysis identifies a number of trends in the data set (canonical variates) that maximally discriminate among the identified groups (in this case, acoustic treatments). CDA derives canonical variates (i.e., linear combinations of the continuous variables) that summarize between-group variation in a similar way to how principal component analysis summarises total variation. Trends in the original variables (routine swimming and fast-start variables) are represented as vectors given by correlations of these variables with the canonical variates (also known as total structure coefficients). These vectors are plotted on the first two canonical axes, together with treatment centroids and their 95% confidence clouds^35^. The strength or importance of each of the original variables in discriminating among groups is displayed graphically as the length and direction of these vectors.

One-factor analysis of variance was used to determine whether the acoustic treatments affected the routine swimming, thigmotaxis or kinematics of the white tailed damselfish. When significant, the nature of the differences was examined using planned comparisons to address four *a priori* hypotheses: (a) does boat noise, regardless of source, affect fish behaviour? (i.e., comparing ambient to grouped mean of the 4 boat noise treatments); (b) is there a difference between fish affected by the playback of 2-stroke engine noise compared to 4-stroke engine noise? (regardless of whether acute or chronic); (c) is there a difference between fish affected by the playback of acute or chronic 2-stroke engine noise? (i.e., 2-stroke acute vs 2-stroke chronic); (d) is there a difference between fish affected by the playback of acute or chronic 4-stroke engine noise? (i.e., 4-stroke acute vs 4-stroke chronic).

To determine the nature and duration of the effect of boat noise on routine swimming, total distance moved was measured in 15 s intervals for the 120 s routine swimming period. This was analysed using a multivariate repeated measures ANOVA, with factor ‘acoustic treatment’ (ambient reef, 2-stroke, 4-stroke), and time (eight 15 s time intervals). One-factor ANOVAs were used to determine the nature of the significant interaction between treatment and time. Data were log_10_ transformed to satisfy the assumptions of the test.

## Results

**Figure 1:**
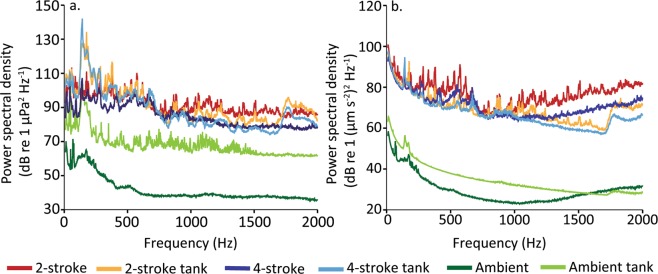
Spectral content of ambient reef and motorboat recordings (2-stroke and 4-stroke outboard engine types) in the field and playback of the recordings within the activity arena measured in both the (**a**) acoustic pressure and (**b**) triaxial particle acceleration acoustic fields. Mean spectral density levels were determined by combining one minute of the four tracks for each treatment.

There was a marked difference between ambient and boat noise power spectra in both the pressure and particle acceleration domains for both the field measurements and recordings made within the experimental arena of the playback of all tracks (Fig. 1a). An examination of the power spectral density of pressure showed that playback tracks preserved the relative pattern of the original tracks between boats and ambient sound across frequencies. However, there was some distortion evident between 100–200 Hz. Particle motion was similar for all boat noise tracks (field and playback) up to 1000 Hz, at which time the playback tracks showed gradually less energy than the field recordings, though the relative difference between motor types was largely maintained (Fig. 1b). Comparing playback of boat passes showed 2-stroke tracks were higher in four out of the five acoustic metrics (Table 3). Two stroke engines produced noise with a higher root-mean-square (RMS), which indicates that the sound wave carried more energy per unit time. In keeping with this finding, 2-stroke engines also had higher peak energy levels, a larger 90% energy envelope, and higher consistency (Table 3). In contrast, acoustic complexity was found to be significantly greater for the 4-stroke than the 2-stroke playback tracks (t_12.5df_ = −5.7, p < 0.001; Table 3). This is because 2-stroke engines produced noise over a broader time period than 4-stroke engines (Fig. S3).

**Table 3:** Acoustic comparisons of playback tracks of 2- and 4-stroke boat passes.

Noise playback	RMS level (30 s) (dB re µPa)	Peak level (dB re µPa)	Consistency (% time over 150 dB re 1 µPa)	90% energy envelope (ms)	ACI
2-stroke	151.74	186.68	30.07	18988.05	11.39
4-stroke	150.18	182.41	18.14	11142.30	12.38

Acoustic metric given are: Mean Root-mean-squared (RMS) sound pressure levels over 30 s, Peak level, Consistency, 90% energy envelope and Acoustic complexity Index (ACI). Ten tracks of 30 s per engine type were analysed in MATLAB 2013a using paPAM analysis package and “Soundecology”(ACI) with a bandwidth of 0–2 kHz.

**Figure 2:**
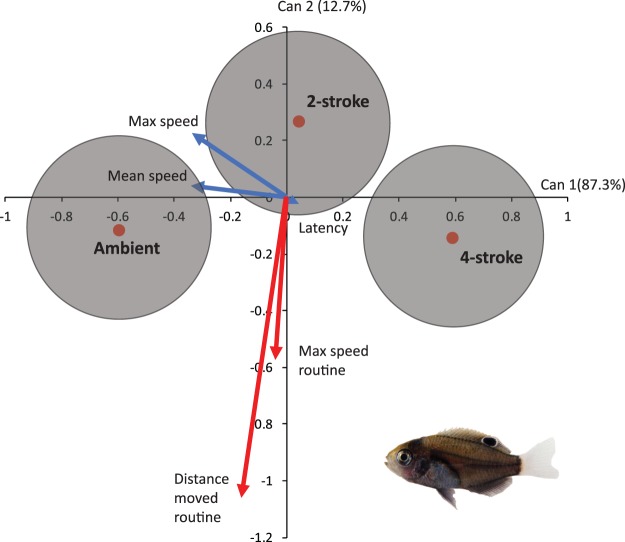
Comparison of the effect of the playback of ambient reef sound or boats with one of two outboard motors types (2- or 4-stroke) on the routine swimming and fast-start kinematics of juvenile whitetail damselfish, *Pomacentrus chrysurus*. The plot (a canonical discriminant analysis bi-plot) summarises how the acoustic treatments affected behaviour as measured by routine swimming (red vectors) and fast-start kinematics (blue vectors). 95% confidence clouds are also given. N = 55 (ambient), 51 (2-stroke), 52 (4-stroke). Photographic credit: M. McCormick.

There was a significant difference in the routine swimming and fast-start response of the white-tail damsel under the three acoustic treatments (MANOVA, Pillai’s Trace 0.230, F_10,304_ = 3.945, P < 0.0001). A CDA displayed the nature of the differences found among treatment centroids (Fig. 2). Fish exposed to ambient and boat noise were differentiated along the first canonical axis, which accounted for 87.3% of the difference among treatments. This axis was principally driven by trends in fast-start kinematics, which indicated that fish under ambient reef sound had higher maximum and average speeds, and a more rapid response to the drop stimulus (i.e., lower response latency) than fish under either boat noise treatment. The greatest difference was between fish exposed to ambient reef sounds compared to 4-stroke boat noise (Fig. 2). In addition, fish exposed to 2-stroke boat noise were differentiated from both the 4-stroke and ambient sound fish based on their routine swimming characteristics, which were the drivers of the second canonical axis (12.7%). Specifically, fish exposed to 2-stroke noise had slower maximum routine swimming speeds (prior to being startled) and were more active, as represented by total distance moved (Fig. 2).

**Figure 3:**
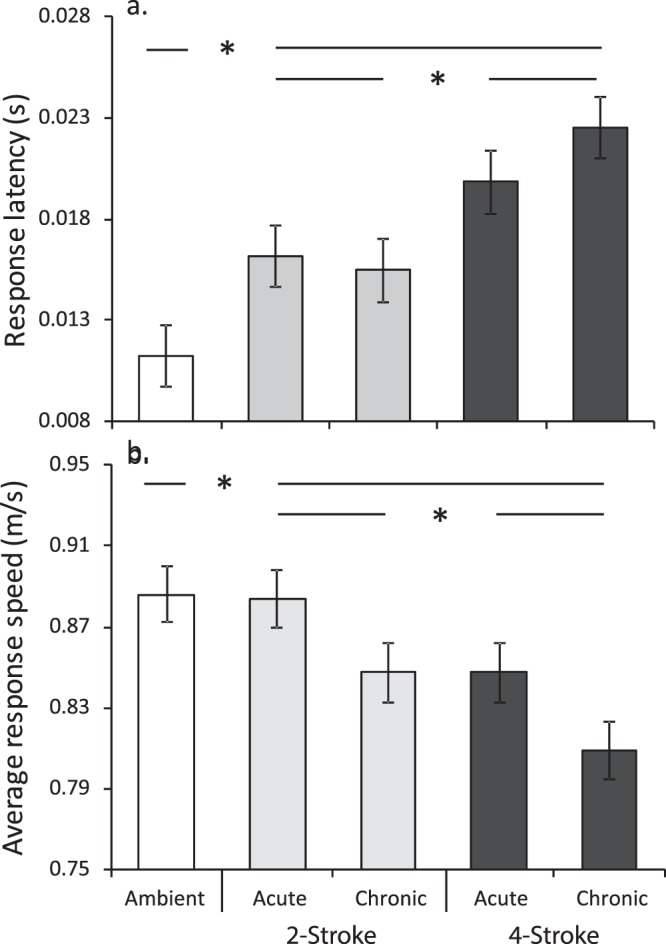
Effect of playback of ambient reef sound (white bars) or one of two outboard motors types (2- or 4-stroke; light grey and dark grey respectively) on the fast-start kinematics of juvenile whitetail damselfish (*Pomacentrus chrysurus*). The kinematic variables (mean ± SE) represented are: (**a**) response latency, and (**b**) average response speed. Boat noise was played either at the same time as the drop stimulus (acute), or for 5 min prior to the drop stimulus (chronic). Data for latency were square-root transformed for analysis and standardised for distance to stimulus, though back-transformed covariate-standardised data are plotted. Results of planned comparisons are superimposed over the bars, with lines between means representing no significant difference, and asterisks representing significant difference between groups. N (left to right) = 55, 54, 51, 49, 52.

Univariate analyses on the fast-start kinematics supported the trends described by CDA. There was a significant difference in the response latency among treatments (F_4,255_ = 54.47, P < 0.0001). Planned comparisons indicated that fish under ambient reef sound had significantly faster response latencies (i.e., they reacted faster) than fish under either boat noise treatment (P < 0.0001), however, fish exposed to 2-stroke noise had faster latencies than fish exposed to 4-stroke engine noise (P = 0.0006, Fig. 3a). There was no effect of whether exposure to boat noise was either chronic or acute (planned comparisons, P > 0.22). Average response speed showed the same, though reverse, trend in significance as latency (Table 3 and Fig. 3b). Planned comparisons found that fish exposed to ambient reef sound exhibited the fastest response speeds compared to fish exposed to the playback of boat noise (P = 0.01), while fish exposed to 2-stroke boat noise had a higher response speed than fish exposed to the playback of 4-stroke boats (P = 0.009, Fig. 3b). In a similar way to latency, average speed was not affected by whether the boat noise exposure was chronic or acute (P > 0.06).

**Figure 4:**
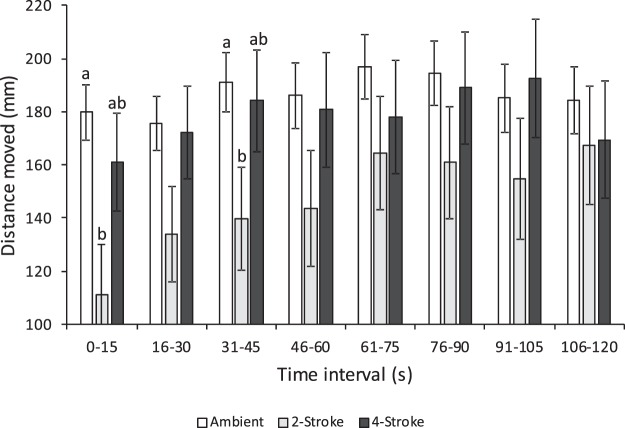
Effect of playback of ambient reef sound (white) or noise from one of two outboard motors types [2-stroke (light grey) or 4-stroke (dark grey)] on the mean distance moved (m ± SE) by juvenile whitetail damselfish (*Pomacentrus chrysurus*) in 15 s intervals from when acoustic treatments were started, after the initial experimental habituation period (prior to being startled and during the ‘routine swimming’ period). Letters represent Tukey’s HSD means groupings within time periods where significant difference among treatments were found. N = 158 (ambient), 51 (2-stroke), 52 (4-stroke).

Acoustic treatment affected the total distance moved during routine swimming, but this effect was influenced by time (acoustic treatment x time: Pillai’s trace = 0.097, F_14,506_ = 1.832, P = 0.032; Fig. 4). There was a significant difference between treatments in the first 15 s, with fish exposed to noise from the playback of 2-stroke powered boats moving less than either fish exposed to ambient or 4-stroke engine noise. This trend continued for the first 45 s, after which there was no significant difference among the treatments. There was no difference in thigmotaxis between acoustic treatments (F_2,258_ = 0.84, P = 0.43).

## Discussion

Few studies have examined the effect of noise from different engine types on fish behaviour, and this is the first study to examine the effect of noise with differing acoustic characteristics on routine behaviour and escape response kinematics. Our study shows that the playback of boat noise had a marked influence on the routine swimming, latency to respond and escape kinematics of a common juvenile damselfish. Moreover, the noise from playbacks that originated from 2- and 4-stroke outboard engines was sufficiently different to result in different impacts on both general space use, activity and escape response. This information forms part of a growing literature that allows us to better understand how fishes are affected by anthropogenically produced noise from different sources (e.g.^36,37^). This is particularly useful to management bodies because noise sources, particularly from vessels, can be modified and managed relatively easily compared to other forms of pollution.

The playback of noise from motorboats affected both the routine swimming and escape response of the white-tailed damsel, but this effect differed depending on the source of the noise. Noise from 2-stroke powered boats affected routine swimming, in particular their activity, as measured by the total distance moved. Fish were less active when in the vicinity of noise from 2-stroke powered boats, compared to ambient reef sound controls. However, this effect was only evident for the first 45 seconds after which routine swimming returned to levels comparable with controls. This result suggests that, at least with respect to routine swimming, fish may be able to habituate to noise over a relatively short time period. Similar potential to habituate to 2-stroke noise was found for another damselfish, the Ambon damselfish (*Pomacentrus amboinensis*), in response to real boat noise in the field. Holmes *et al*.^38^ found that the level of boldness exhibited by juvenile fish on habitat patches was immediately reduced in the presence of 2-stroke noise but recovered to pre-noise levels after 20 min of continuous motorboat noise. It is unclear why the time frames of habituation to noise are so different between these studies, but it may be due to factors including the artificiality of experiments, species-specific differences or difference in the metrics of activity. These differences between studies, and between engine types within our study, emphasise that the capacity of fishes to habituate to sound is a field that warrants further research^39^.

In contrast, noise from 4-stroke powered boats had little effect on routine swimming, and instead had a major influence on the fast-start escape response. In particular, their speed to mount a fast-start response (latency) was over three-times slower in the presence of 4-stroke noise compared to ambient reef sound controls. This compared to a doubling of speed to initiate a fast-start in the presence of noise from 2-stroke powered boats. This slowing of response times has the potential to greatly affect survival of an aware prey in response to a predator strike. A recent field study that assessed a broad range of performance criteria for newly settled damselfish found that latency to respond to a drop stimulus best predicted survival in the field out of 18 measured variables^22^.

To date, only one previous study has compared the effects of the noise from 2- and 4-stroke powered boats on fishes. McCormick *et al*.^18^ examined the ability of a newly settled damselfish to assess and respond to risk in the presence of noise from passing 2- or 4-stroke boats over a shallow reef. Risk assessment was quantified as the ability of fish to respond with an antipredator response to odours released through the damage of conspecifics, while their ability to mount an effective startle response was assessed with an *in situ* looming stimulus test. In perciforms, which make up the majority of coral reef fishes, damage to the epidermis releases chemicals that elicit an antipredator response in conspecifics and closely related heterospecifics (e.g.^40^), which is similar to the chemical alarm odours released by freshwater ostariophysan fishes upon damage of club cells within the epidermis^41^. For both the odour and startle tests, fish were found to be incapable of mounting effective antipredator responses in the presence of noise from passing boats powered by 2-stroke outboard engines. Moreover, in the presence of 2-stroke noise, fish actually elicited a feeding response to the alarm odour, which contrasted to their risk averse response to alarm odours in the presence of 4-stroke engine noise. Given that alarm odours are only released upon damage of the skin of conspecifics they represent a reliable indicator of a nearby threat^40,42–44^, and so the feeding response found represents a maladaptive response. These findings, together with other studies in the laboratory that used playback of boat, ship or pile driving noise (eels^45^; sea bass^46^), add weight to our findings of a marked effect of engine noise on the startle response of fishes.

Our experiment also found that the decrement in the efficacy of the fast-start response when exposed to the playback of noise from either 2- or 4-stroke engines was not due to the initial shock of an abrupt change in the soundscape. There was no difference in the fast-start kinematics between fish startled immediately upon the imposition of boat noise, and those that had been exposed to noise for up to 6 minutes prior to the drop-stimulus. This suggests that the noises affect the sensory system of the small fish in such a way that they cause an ongoing distraction over an ecologically relevant time frame. While routine swimming activity appeared to either not be affected (4-stroke noise) or recover quickly (2-stroke noise), the sensory inputs that govern the initiation (i.e., latency) and speed of the C-start burst response could not adjust to the discombobulating effects of noise. Chan *et al*.^47^ experimentally examined the fright response of hermit crabs (*Coenobita clypeatus*) under the influence of boat noise playback and concluded that distraction was the most likely mechanism underlying this response.

How fishes are affected by noise will depend on the frequency and intensity (dB) of the noise in relation to their capacity to hear sound. Frequency spectra in both the pressure and particle acceleration domains were consistently higher for the two-stroke noise compared to noise from 4-stroke engines. This made for a greater sound exposure in both domains for fish exposed to noise from 2-stroke engines. Moreover, the acoustic complexity was higher for noise from passing boats powered by 4-stroke engines, which is because boats powered by 2-stroke engines produced noise over a broader range of frequencies for a longer period of time during a boat pass. Interestingly, while both engine types produce noise spectra that are markedly different from ambient reef noise, the relatively subtle differences between engine-noise types has substantial influences on the ability of fishes in the vicinity to use other sensory cues, such as vision and smell^17,18^. The impacts of the subtle acoustic differences between engine types found in the present study suggest that other types of marine noise pollution (e.g., different vessel types, underwater turbines, seismic surveys^48,49^; are likely to have different effects on marine organisms depending upon their frequency spectra, and how they change through time.

Our study underscores the impact of anthropogenic noise on important aspects of the dynamics of fish populations. We have limited knowledge as to how long lasting these effects are, and the extent to which fish can habituate to this source of pollution^14^. The role that the auditory history of an individual plays in its physiological and behavioural response to recurrent and new noise sources is largely unknown (e.g.^50^). The spatial scale of these effects, the extent to which fish may use topography as barriers to sound, and the importance of the duty cycle of noise all warrant further research. The current study is supported by less-detailed field studies and emphasises the impact noise may have on a fundamental behavioural response – the avoidance of risk. Our findings underscore that all marine noise will not have the same impact on fishes, and this gives managers tools whereby they can actively reduce a key stressor on endangered or prioritised communities they decide to protect.

## Supplementary information

Suuplementary information

## Data Availability

Data are available at: 10.25903/5b8cac554a2b0.
