# Supplementary material for: Effects of boat noise on fish fast-start escape response depend on engine type
Source: Sci Rep. 2019 Apr 25;9:6554. doi: 10.1038/s41598-019-43099-5 (PMC6484016; doi:10.1038/s41598-019-43099-5)
Supplement: Supplementary file 1 — Suuplementary information [file 41598_2019_43099_MOESM1_ESM.pdf]

Supplementary files

Effects of boat noise on fish fast-start escape response depend on engine type

Mark I. McCormick, Eric P. Fakan, Sophie L. Nedelec, Bridie J. M. Allan

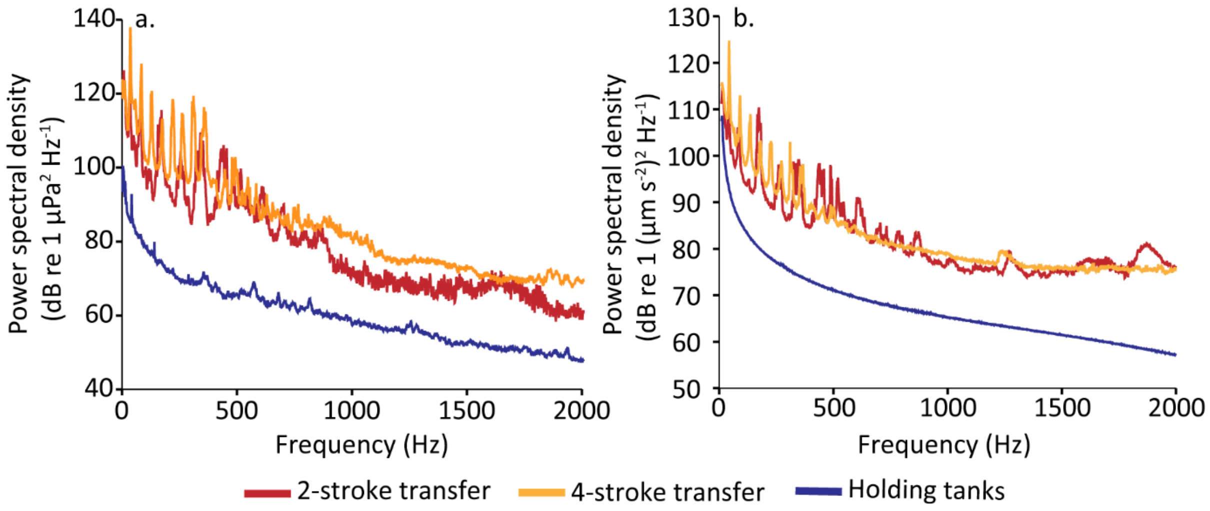

Figure S1. Mean power spectral density of acoustic conditions of (a) sound pressure and (b) particle acceleration for transferring light trap fishes in 60 L nally bins from 2- and 4-stroke engines and within the 25 L holding tanks. Mean spectral density levels were determined by a one minute recording during boat transfers and a two-minute recording of the housing tanks with a submerged inflow.

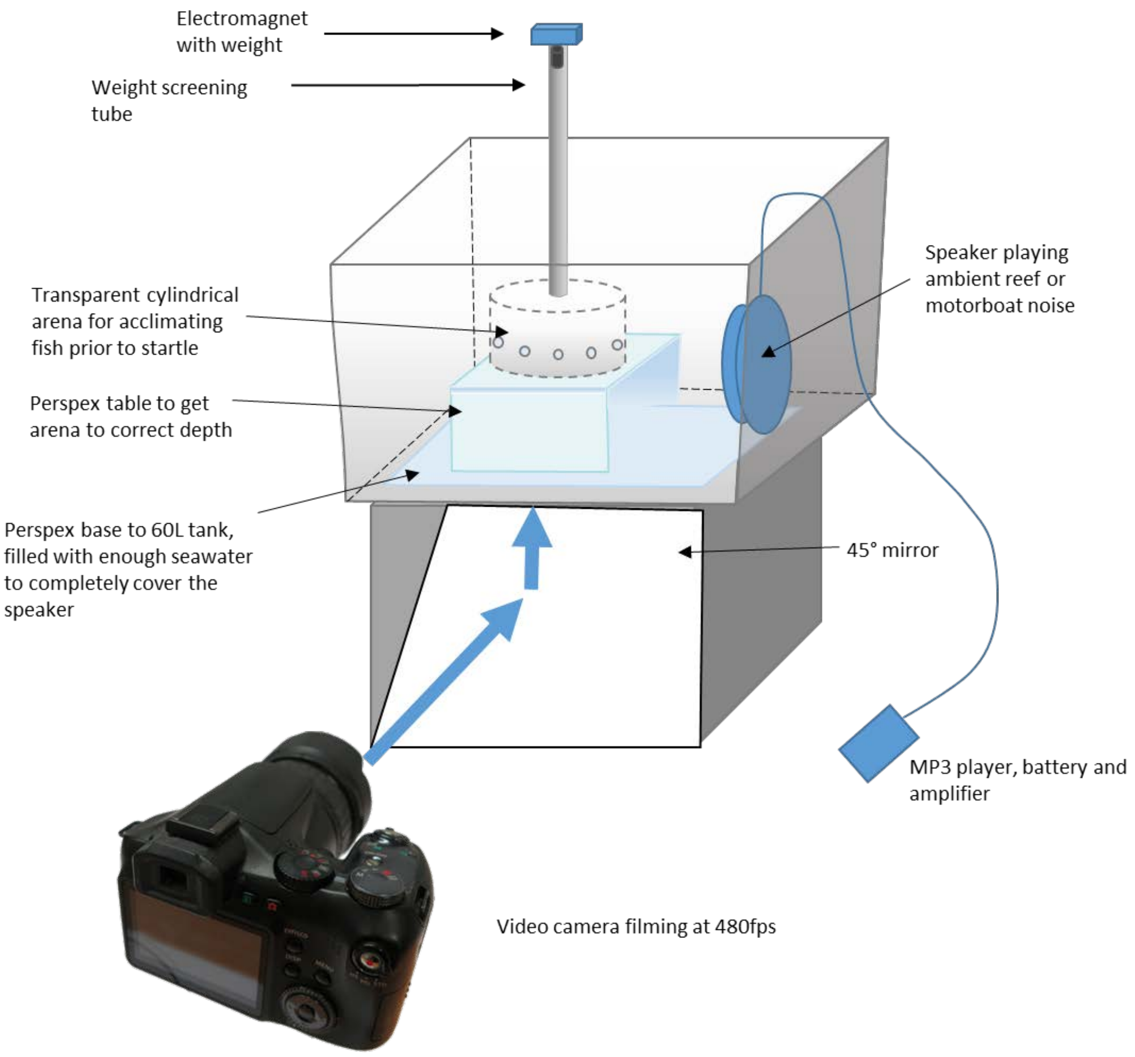

Figure S2. Fast start apparatus. Fish were placed into the arena to habituate for 5 min under ambient reef noise played through the speaker. Fish were then either exposed to ambient reef, or noise from motorboats powered by either 2- or 4-stroke 30hp outboard motors for 5 min. Fish were then startled with a drop stimulus (weight) while in the presence of one of the three noise recordings.

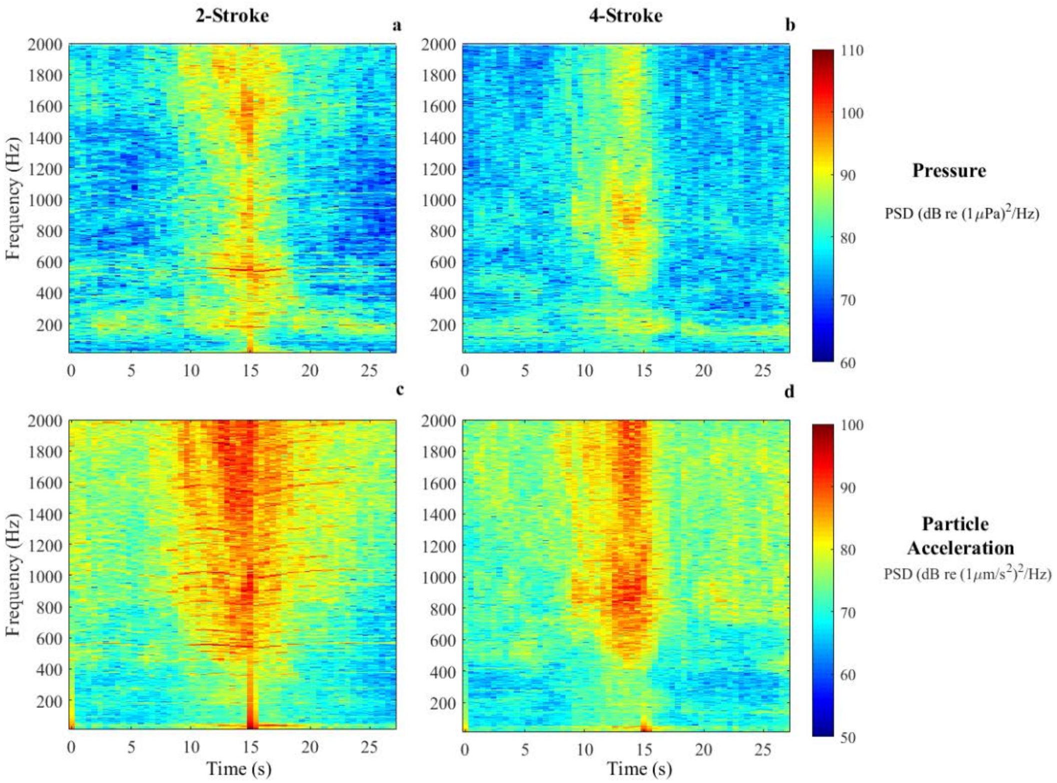

Figure S3. *In situ* spectrograms of a single boat pass of 2- (a) and 4-stroke (b) engines in the pressure domain as well as their triaxial particle acceleration, (c) and (d) respectively. Fast-Fourier Transformation = sampling rate (44100 Hz), Hamming windows, bandwidth between 0-2000 Hz. Passes were centred on the position of greatest noise intensity.
